# Pose Determination of the Disc Cutter Holder of Shield Machine Based on Monocular Vision

**DOI:** 10.3390/s22020467

**Published:** 2022-01-08

**Authors:** Dandan Peng, Guoli Zhu, Dailin Zhang, Zhe Xie, Rui Liu, Jinlong Hu, Yang Liu

**Affiliations:** School of Mechanical Science and Engineering, Huazhong University of Science and Technology, Wuhan 430074, China; pdd@hust.edu.cn (D.P.); m202070528@hust.edu.cn (Z.X.); lrui@hust.edu.cn (R.L.); d202180301@hust.edu.cn (J.H.); d201780247@hust.edu.cn (Y.L.)

**Keywords:** monocular vision, disc cutter holder, OPnP, distance transformation, pose estimation

## Abstract

The visual measurement system plays a vital role in the disc cutter changing robot of the shield machine, and its accuracy directly determines the success rate of the disc cutter grasping. However, the actual industrial environment with strong noise brings a great challenge to the pose measurement methods. The existing methods are difficult to meet the required accuracy of pose measurement based on machine vision under the disc cutter changing conditions. To solve this problem, we propose a monocular visual pose measurement method consisting of the high precision optimal solution to the PnP problem (OPnP) method and the highly robust distance matching (DM) method. First, the OPnP method is used to calculate the rough pose of the shield machine’s cutter holder, and then the DM method is used to measure its pose accurately. Simulation results show that the proposed monocular measurement method has better accuracy and robustness than the several mainstream PnP methods. The experimental results also show that the maximum error of the proposed method is 0.28° in the direction of rotation and 0.32 mm in the direction of translation, which can meet the measurement accuracy requirement of the vision system of the disc cutter changing robot in practical engineering application.

## 1. Introduction

A shield tunneling machine [1] is a special construction machine for tunneling, which has been widely used in subway, railway, highway, and other tunnel projects. With the significant improvement of human construction level and the vigorous development of the tunnel industry, intelligent tunneling equipment has become a development trend. The existing shield tunneling machine, which has been widely used in tunnel construction, is semi-automatic and needs both manual operation and automation execution, such as the changing of the worn disc cutter on the cutter head still needs human to do. Furthermore, the disc cutter consumption is so huge during the construction process that the disc cutter needs to be changed frequently. However, the environment for disc cutter changing is generally quite harsh, especially in mud balance shield machines of high humidity and high pressure, making the manual operation mode a significant potential safety hazard. For example, there have been many disc cutter changing accidents at home and abroad. The disc cutter detection and changing cost more than 10% of the construction period of the tunnel, so it is necessary to develop a disc cutter changing robot to realize the automatic operation. The robot’s vision system, which is used to obtain the disc cutter pose, should have high measuring accuracy and robustness to help the robot accurately grasp the disc cutter.

The visual measurement method includes measurement based on cooperative target [2,3] and that based on non-cooperative target [4], the former is to locate the object by artificial markers fixed on it, and the latter is generally to locate the object by its features. The artificial marks processed on the disc cutter or its holder are easy to be worn; thus, the non-cooperative target method is more suitable for the actual situation. Usually, some prominent geometric features with simple structure and easy recognition are extracted from the object for positioning, such as point [5], straight line [6], circle [7], etc. By using the corner points on the inside contour of the cutter holder, feature points are extracted to estimate the pose of the cutter holder. The feature extraction can be realized by the visual measurement system after image acquisition and processing of the object. According to the dimension information, visual measurement can also be divided into monocular measurement [8,9] and multi-ocular measurement [10]. Monocular measurement is simple in structure, light in weight and low in cost, but it is difficult to obtain the depth information of objects. Multi-ocular measurement can obtain the depth information of objects, which is often used in the 3D reconstruction. Compared with multi-ocular measurement, monocular measurement has no image matching process and only needs a single image to obtain the pose, and has higher positioning efficiency [11]. The actual disc cutter changing space is very narrow, and the vision system is installed on the end actuator of the disc cutter changing robot. The lower the load of the end actuator, the more dexterous the operation [12]. In addition, the installation space of the end actuator is limited, and the small volume of the monocular measurement system is easier to install. Therefore, the monocular measurement mode is chosen forthe visual system in the disc cutter changing robot.

Pose measurement based on monocular vision is also called the Perspective-n-Point (PnP) problem [13]. Currently, there are many methods proposed to solve this problem. Among these, the DLT [14,15] method is classical and efficient but vulnerable to noise interference and has poor robustness. Li et al. have proposed an RPnP [16] method, which can always present stable calculation results regardless of the number of feature points, but it cannot get a unique solution when using the least square error, and its solving accuracy needs to be improved. The EPnP [17,18,19] is a non-iterative method that uses virtual points to indirectly solve the pose parameters of the target, thus reducing its computational complexity, and it also has good anti-noise compared with other PnP methods. However, when the number of feature points is small, its solving accuracy and robustness are relatively low. The basic idea of the ASPnP [20] and the OPnP [21,22] method is to formulate the PnP problem into a functional minimization problem and retrieve all its stationary points using the Gröbner basis technique. These two methods have high accuracy and robustness and are the best PnP methods so far. 

The existing PnP methods can guarantee high accuracy when the noise is slight; otherwise, the accuracy is low. Since the image of the cutter holder collected in the actual industrial environment contains strong noise, a method with good robustness to solve the pose of the cutter holder is needed. The distance matching method [23] is a template matching method that calculates the distance between the template and the measured object image to solve the object pose, which is robust but depends on the initial pose of the measured object. The key part of the distance matching method is the distance transform. Distance transform [24,25] is an operation for binary image, which can be used in the template matching method to estimate target pose [26]. Different distance measures will produce different transformation results, among which the most commonly used distances are Euclidean distance [27], Manhattan distance [28], and chamfer distance [29]. Euclidean distance represents the exact distance between the template and the object image, and the other distances are approximate expressions of Euclidean distance [30]. The accuracy of the distance matching method depends on the accuracy of the given initial pose, and it is widely used to solve the pose based on contour features [31]. Hu et al. [32] introduced a method to estimate the pose of the pipe, which firstly used a template matching method to estimate the pipe’s initial pose, then used the least-squares method to obtain a more accurate pipe pose. A combination of the initial pose estimation method and the precise positioning method can be used to obtain the object pose.

In this paper, we propose the OPnP+DM method to obtain the cutter holder pose of the shield machine. First, before the pose measurement, the pose template library of the cutter holder is established offline. Second, the image coordinates of the feature points on the cutter holder surface can be obtained by the image processing method. We can use these feature points to solve the rough pose of the cutter holder by the OPnP method. Third, we take this initial pose as input and use the distance matching method to solve the pose accurately. Meanwhile, the distance between the collected image of the cutter holder and the template in the established pose template library is calculated. The template corresponding to the minimum distance is selected, and its corresponding pose is used as the final estimated pose of the cutter holder.

The innovations of this paper include:

(1) The OPnP+DM method, which consists of the optimal accuracy OPnP method and the highly robust distance matching method, takes advantage of the two methods.
(2) The OPnP+DM method extracts the characteristics of the cutter holder without processing the artificial marks, which reduces the labor and improves the stability of visual measurement.
(3) The step acceleration method is proposed to speed up the search of the optimal pose in the distance matching method.
(4) The proposed OPnP+DM method can achieve a positioning accuracy of 1 mm required by the vision measurement system of the disc cutter changing robot under the condition of strong noise, which cannot be achieved by the PnP methods.

The rest of this paper is organized as follows. Section 2 states the pose estimation method of the cutter holder. In Section 3, the simulation results are used to prove the accuracy and robustness of our method. In Section 4, experiments are implemented to test the feasibility of the practical engineering application of our method. In Section 5, the conclusions are given.

## 2. Proposed Method

**Figure 1:**
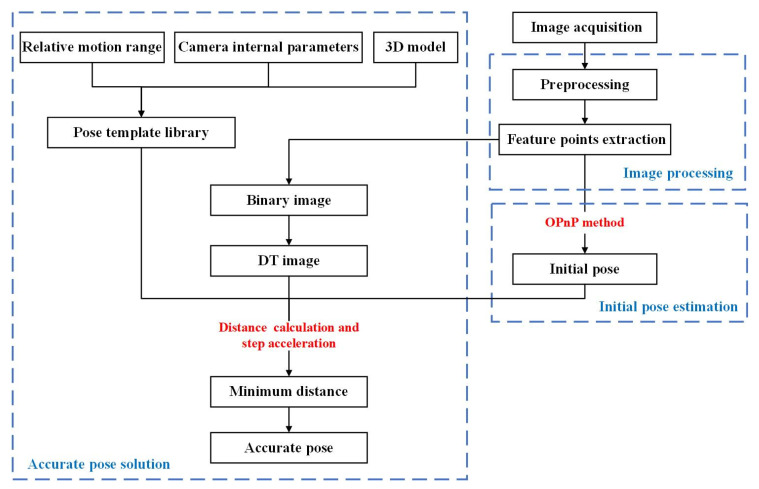
Flow diagram of the pose estimation method.

The flow diagram of the pose estimation method based on monocular vision proposed in this paper is shown in Figure 1. We first calibrate the monocular system to obtain the internal parameter matrix of the camera, which is used to establish the pose template library offline. Then, we use the calibrated camera to collect the cutter holder images. The images are preprocessed, and the image processing method is further designed to extract the required image features. According to the extracted features, the initial pose estimation method is used to obtain the initial pose of the cutter holder. We take the initial pose as input and find it in the existing pose template library. With the initial pose as the center, some templates are selected from the pose template library by setting a step size and a large enough search range. By substituting the image coordinates of the template into the DT image, the distances between templates and the actual cutter holder are obtained one by one, and the template corresponding to the minimum distance is screened out. Further, we can change the step length until the estimated pose of the cutter holder meets the actual measurement accuracy requirements. Image processing, initial pose estimation, and accurate pose solution in the proposed method are detailed as follows.

### 2.1. Image Processing

**Figure 2:**
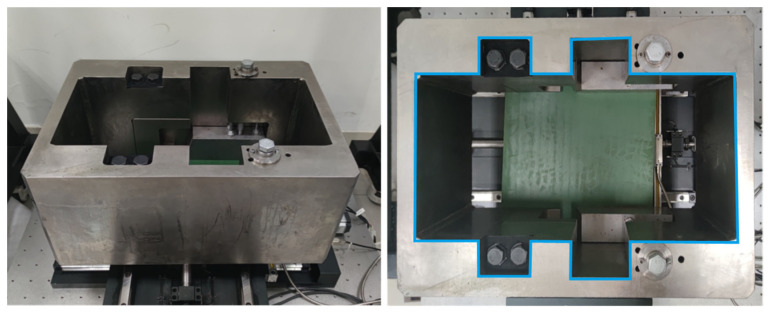
The physical photo of the cutter holder. The blue curve on the surface of the cutter holder on the right is its inside contour.

It is necessary to extract the salient features of the cutter holder to estimate its pose accurately. Linear features and point features are the most commonly used geometric information in robot visual positioning. The real object of the new-type cutter holder, which needs to be measured in this paper, is shown in Figure 2, and its machining accuracy is 0.2 mm. The marked blue contour on its surface marked in Figure 2 is its inside contour feature, and there are 20 feature corner points on the inside contour of the cutter holder surface. It is difficult to extract these feature points directly because of the wear and corrosion on the surface of the cutter holder caused by the extended time working of shield tunneling. In addition, other structures on the cutter holder surface, such as fastening bolts and mounting holes, will also interfere with the extraction of feature points. Therefore, we can first extract the complete inside contour of the cutter holder and then further obtain the feature points on it.

The whole image processing process of the feature points detection can be divided into preprocessing block and feature points extraction block. The input of the preprocessing block is the collected cutter holder image by the vision system, and the output is the binary image containing the region of interest of the cutter holder. In the preprocessing block, the median filtering method is used to filter the collected cutter holder image. Then, the correlation template matching method [33] is used to obtain the region of interest (ROI) containing the inside contour of the cutter holder by using the template image stored in advance. Correlation template matching is also a method based on gray features, which uses a normalized cross-correlation matching method based on row vectors. It is much faster and adapts to linear lighting changes better than the gray template matching method. In the method, only the ROI of the images are processed, which significantly improves the image processing efficiency. There is a gray difference between the cutter and its holder, so we can use the global threshold segmentation method to obtain the binarized image of the ROI. The input of the feature points extraction block is the binary image obtained by the preprocessing block, and its output is the image coordinates of the 20 feature points on the inside contour. In the feature points extraction block, the subpixel closed contour curves of the binary image are extracted, their length is sorted, and the longest inside contour of the cutter holder is extracted. We divided the inside contour into predefined shapes, such as line segments, arcs, ellipses, and other parts. Collinear processing and line fitting are carried out on them successively, and finally, 20 feature lines are obtained on the surface of the cutter holder. The intersection points of adjacent feature lines are solved to obtain the image coordinates of the 20 feature corner points, which are used to solve the cutter holder pose.

### 2.2. Initial Pose Estimation

We adopt the OPnP method with better accuracy and robustness among the presented PnP methods as the initial pose estimation method. The OPnP method is an efficient pose solution method considering Gaussian noise. It parameterizes the rotation matrix using non-unit quaternion, which contributes to getting a system of polynomial equations constituted of the globally optimized objective function. Finally, the Gröner basis [34] is used to solve the polynomial equations and obtain the final pose.

Given n 3D reference points $q_{i}={\left[x_{i}\;y_{i}\;z_{i}\right]}^{T}$, *i* = 1, 2, …, *n*, and their corresponding image point $p_{i}={\left[u_{i}\;v_{i}\;1\right]}^{T}$. The PnP problem aims to obtain the object pose, which contains the rotation matrix **R** and the translation vector **t**. The perspective imaging equation reads

(1) $$\lambda_{i}p_{i}=Rq_{i}+t,\;i=1,2,\cdots ,n,$$

where *λ_i_* denotes the depth factor of the *i*-th point. Vectors, scalars, and matrices are denoted by using bold lowercase letters, plain lowercase letters, and capital letters, respectively.

Set $s=a^{2}+b^{2}+c^{2}+d^{2}$, and **R** in OPnP method is represented by quaternion as follows

(2) $$R=\frac{1}{s}\left[\begin{array}{ccc} a^{2}+b^{2}-c^{2}-d^{2} & 2bc-2ad & 2bd+2ac\\ 2bc+2ad & a^{2}-b^{2}+c^{2}-d^{2} & 2cd-2ab\\ 2bd-2ac & 2cd+2ab & a^{2}-b^{2}-c^{2}+d^{2} \end{array}\right],$$

where *a*, *b*, *c*, *d* are unknown parameters. For any nonzero value *k*, **R** (*a*, *b*, *c*, *d*) = **R** (*ka*, *kb*, *kc*, *kd*). We can use the reciprocal of the average depth $s=\frac{1}{\frac{1}{n}\sum_{i=1}^{n}\lambda_{i}}=\frac{1}{\bar{\lambda }}$ to fix the value of *k*. Multiply both sides of Equation (1) by $\frac{1}{\bar{\lambda }}$ to get the following equation:

(3) $${\hat{\lambda }}_{i}\left[\begin{array}{c} u_{i}\\ v_{i}\\ 1 \end{array}\right]=\left[\begin{array}{c} r_{1}^{T}\\ r_{2}^{T}\\ r_{3}^{T} \end{array}\right]q_{i}+\left[\begin{array}{c} {\hat{t}}_{1}\\ {\hat{t}}_{2}\\ {\hat{t}}_{3} \end{array}\right],i=1,2,\cdots ,n,$$

in which ${\hat{\lambda }}_{i}=\frac{\lambda_{i}}{\bar{\lambda }}$, $\;{\left[\begin{array}{ccc} {\hat{t}}_{1} & {\hat{t}}_{2} & {\hat{t}}_{3} \end{array}\right]}^{T}=\frac{1}{\lambda }t$, and

(4) $$\left[\begin{array}{c} r_{1}^{T}\\ r_{2}^{T}\\ r_{3}^{T} \end{array}\right]=\left[\begin{array}{ccc} a^{2}+b^{2}-c^{2}-d^{2} & 2bc-2ad & 2bd+2ac\\ 2bc+2ad & a^{2}-b^{2}+c^{2}-d^{2} & 2cd-2ab\\ 2bd-2ac & 2cd+2ab & a^{2}-b^{2}-c^{2}+d^{2} \end{array}\right],$$

From Equation (3) we know that

(5) $${\hat{\lambda }}_{i}=r_{3}^{T}q_{i}+{\hat{t}}_{3},\;i=1,2,\cdots ,n.$$

It is known that

(6) $$\sum_{i=1}^{n}{\hat{\lambda }}_{i}=\frac{\sum_{i=1}^{n}\lambda_{i}}{\bar{\lambda }}=n\frac{\sum_{i=1}^{n}\lambda_{i}}{\sum_{i=1}^{n}\lambda_{i}}=n.$$

By summing both sides of Equation (5) and then plugging the result into Equation (6), we have

(7) $${\hat{t}}_{3}=1-r_{3}^{T}(\frac{1}{n}\sum_{i=1}^{n}q_{i})=1-r_{3}^{T}\bar{q},$$

where $\bar{q}$ represents the centroid of the 3D points. Then, we plug ${\hat{\lambda }}_{i}=1+r_{3}^{T}(q_{i}-\bar{q})=1+r_{3}^{T}{\tilde{q}}_{i}$ into Equation (3) and obtain the following equation

(8) $$(1+r_{3}^{T}{\tilde{q}}_{i})\left[\begin{array}{c} u_{i}\\ v_{i} \end{array}\right]=\left[\begin{array}{c} r_{1}^{T}\\ r_{2}^{T} \end{array}\right]q_{i}+\left[\begin{array}{c} {\hat{t}}_{1}\\ {\hat{t}}_{2} \end{array}\right],i=1,2,\cdots ,n.$$

The above results are calculated in the ideal state, but we should consider the effect of actual noise. We can take the following minimum sum of the squared error as our cost function

(9) $$\underset{a,b,c,d,{\hat{t}}_{1},{\hat{t}}_{2}}{\min}\;\sum_{i=1}^{n}{\left[(1+r_{3}^{T}{\tilde{q}}_{i})u_{i}-r_{1}^{T}q_{i}-{\hat{t}}_{1}\right]}^{2}+\sum_{i=1}^{n}{\left[(1+r_{3}^{T}{\tilde{q}}_{i})v_{i}-r_{2}^{T}q_{i}-{\hat{t}}_{2}\right]}^{2}.$$

The accuracy of this minimum is very close to the minimal reprojection error. Before solving Equation (9), we can easily get that

(10) $$\begin{array}{l} {\hat{t}}_{1}=\bar{u}+r_{3}^{T}(\frac{1}{n}\sum_{i=1}^{n}u_{i}{\tilde{q}}_{i})-r_{1}^{T}\bar{q},\\ {\hat{t}}_{2}=\bar{v}+r_{3}^{T}(\frac{1}{n}\sum_{i=1}^{n}v_{i}{\tilde{q}}_{i})-r_{2}^{T}\bar{q} \end{array}$$

in which ${\left[\bar{u},\bar{v}\right]}^{T}$ is the centroid of the image points in the normalized image coordinate system.

Set $\alpha ={\left[1,a^{2},ab,ac,ad,b^{2},bc,bd,c^{2},cd,d^{2}\right]}^{T}$, according to Equations (9) and (10), we can rewrite the cost function into the following form

(11) $$\underset{a,b,c,d}{\min}\;f(a,b,c,d)={\left\|M\alpha \right\|}_{2}^{2}=\alpha^{T}M^{T}M\alpha ,$$

where *M* is a 2n × 11 matrix, and it does not contain any trigonometric function nor constraint. For Equation (11), take partial derivatives of *a*, *b*, *c*, and *d* respectively, we have

(12) $$\frac{\partial f}{\partial a}=0,\;\frac{\partial f}{\partial b}=0,\;\frac{\partial f}{\partial c}=0,\;\frac{\partial f}{\partial d}=0,$$

which compose four three-degree polynomials with respect to *a*, *b*, *c*, *d*.

The Gröbner basis method is used to solve the Equation (12), and the polynomial system has at most 81 solutions. We can get at most 40 effective solutions excluding zero and symmetric solutions. The OPnP method is quite accurate even with a few point correspondences and its computational time keeps almost unchanged as the point number increases [21].

### 2.3. Accurate Pose Solution

#### 2.3.1. The Distance Matching Method

**Figure 3:**
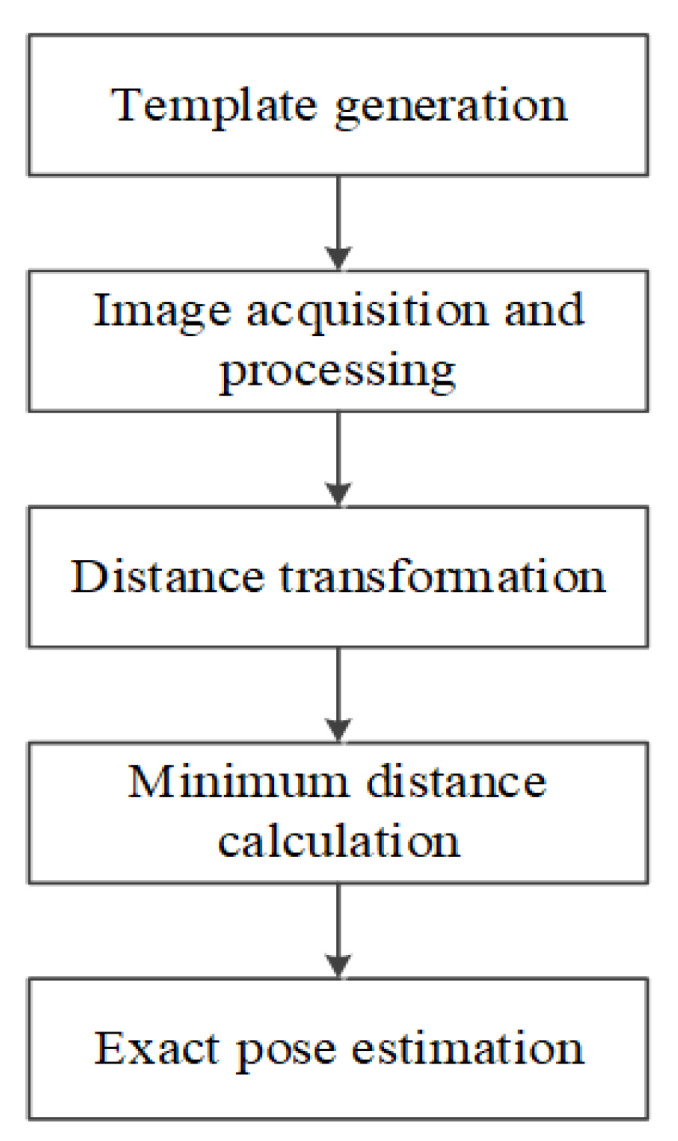
Flowchart of distance matching method.

After estimating the initial pose of the cutter holder, we use the distance matching (DM) method to obtain its accurate pose further. The essence of the DM method is a template matching method based on distance. Many scholars have studied object recognition [26] and pose estimation [31] based on the DM method. However, they all extracted contour features of the object when using this method. In order to simplify the calculation, we only extract a limited number of feature points when using the DM method to estimate the cutter holder pose. Moreover, the DM method relies on the initial pose obtained by the above OPnP method. The DM method is illustrated in Figure 3. First, the pose template library of the cutter holder is established, and then distances between the collected cutter holder image and the templates in the library are solved. Finally, the template corresponding to the minimum distance is screened out, and its pose is taken as the final estimated pose of the actual cutter holder.

The first step of the DM method is to build the pose template library offline. We place the origin of the workpiece coordinate system in the center of the cutter holder surface. The *Z*-axis of the coordinate system is perpendicular to the cutter holder surface, and the *X*-axes and *Y*-axes are respectively parallel to the long and short edges of the outer rectangular contour of the cutter holder. Assuming that the workpiece coordinate system coincides with the world coordinate system, the world coordinate system changes with the workpiece. Therefore, the relative pose of the world coordinate system and the camera coordinate system is the required cutter holder pose. The monocular measurement system is fixed on the end-effector of the disc cutter changing robot and moves with the robot in a limited space. We change the pose of the cutter holder in the space with the step size of 0.1° in the rotation direction and 0.1 mm in the translation direction. In each pose state, according to the 3D model of the cutter holder and the camera projection model, the corresponding image coordinates of the selected 20 feature points on the surface of the cutter holder can be obtained. Each template in the library is represented by the image coordinates of the 20 feature points, corresponding to a pose of the cutter holder in the camera coordinate system. These templates constitute the pose template library of the cutter holder, which significantly reduces the storage space compared with the direct storage of the whole image, even though its number is enormous. Although establishing a pose template library takes a long time, it is offline and does not occupy real-time pose estimation.

**Figure 4:**
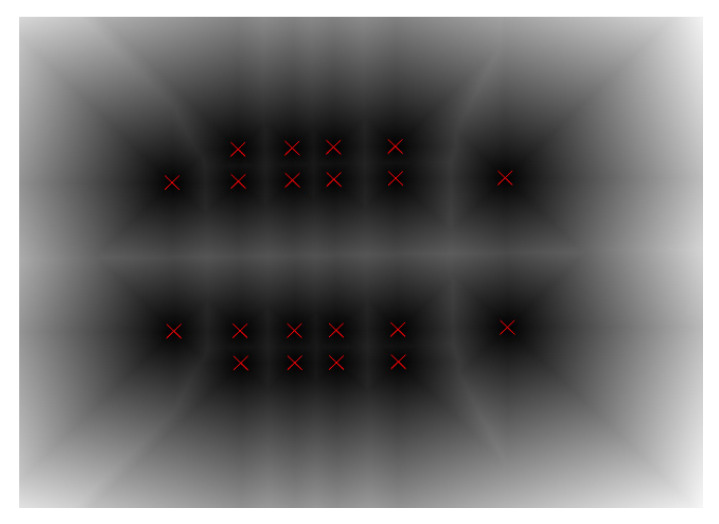
DT image. The distances are gray-level coded: the larger the distance the lighter the tone. The red cross are the feature points of the cutter holder.

The operation converting a binary image to an approximate distance image is called a distance transformation (DT), which can produce a grayscale image. The grayscale value of each pixel is the distance between the pixel and the nearest background pixel. The Euclidean DT image is obtained by distance transformation of the binary image containing only 20 feature points of the cutter holder, as shown in Figure 4. At this point, background pixels are the 20 feature points on the binary image. By substituting the image coordinates of 20 feature points of any template in the pose template library into the distance image, 20 distance values *d_i_* can be obtained, and solve their root mean square value as the distance between the cutter holder and the template, which can be expressed by Equation (13),

(13) $$D=\sqrt{\frac{\sum_{i=1}^{N}d_{i}{}^{2}}{N}},\;i=1,2,\ldots ,N,$$

where *N* is the number of feature points and the value is 20. The template corresponding to the minimum distance in the pose template library is obtained, and its corresponding pose is taken as the estimated pose of the actual cutter holder.

#### 2.3.2. The Step-Accelerating Method

**Figure 5:**
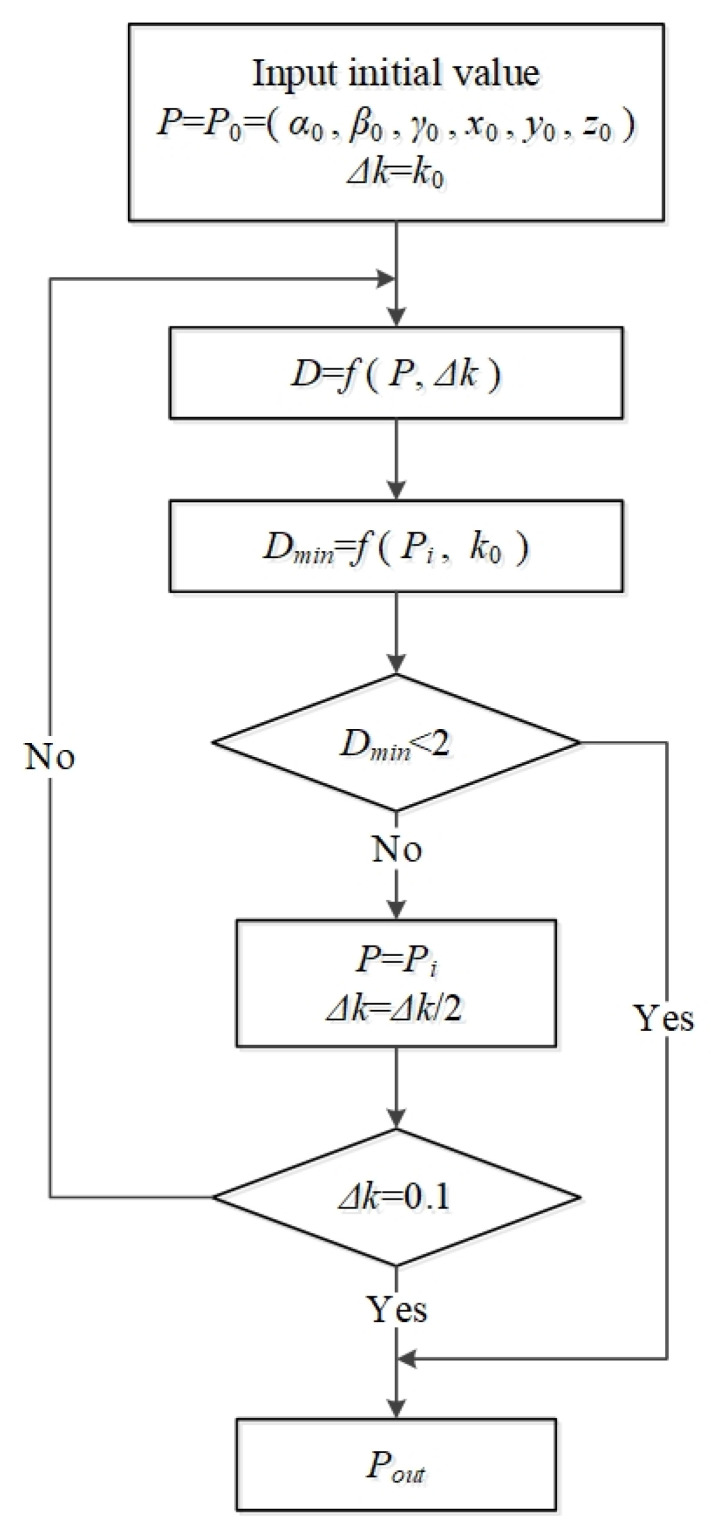
The structural block diagram of the step acceleration process.

To speed up the efficiency of the DM method, we design a step-accelerating method. The structural block diagram of the step acceleration process is shown in Figure 5.

First, we set the pose variable as *p* = (*α*, *γ*, *β*, *x*, *y*, *z*), step size variable of each degree of freedom as Δ*k*. The initial value of variable *p* is *P*_0_ = (*α*_0_, *γ*_0_, *β*_0_, *x*_0_, *y*_0_, *z*_0_), and the initial value of Δ*k* is *k*_0_. In the following experiments, we set the value of *k*_0_ in the DM method as 3.2 according to the initial pose accuracy. Moreover, the six degrees of freedom range are [*α* − 2Δ*k*, *α* + 2Δ*k*], [*γ* − 2Δ*k*, *γ* + 2Δ*k*], [*β* − 2Δ*k*, *β* + 2Δ*k*], [*x* − 2Δ*k*, *x* + 2Δ*k*], [*y* − 2Δ*k*, *y* + 2Δ*k*], [*z* − 2Δ*k*, *z* + 2Δ*k*] respectively. Second, by substituting initial values *P*_0_ and *k*_0_, the pose *P*_1_ = (*α*_1_, *γ*_1_, *β*_1_, *x*_1_, *y*_1_, *z*_1_) corresponding to the minimum distance is obtained by the DM method. The range is further changed to [*α*_1_ − 2*k*_0_, *α*_1_ + 2*k*_0_], [*γ*_1_ − 2*k*_0_, *γ*_1_ + 2*k*_0_], [*β*_1_ − 2 *k*_0_, *β*_1_ + 2*k*_0_], [*x*_1_ − 2*k*_0_, *x*_1_ + 2*k*_0_], [*y*_1_ − 2*k*_0_, *y*_1_ + 2*k*_0_], [*z*_1_ − 2*k*_0_, *z*_1_ + 2*k*_0_]. Third, we can repeat the second step process until the minimum distance is reduced to a constant value *D_min_*, and the pose *P_i_* = (*α_i_*, *γ_i_*, *β_i_*, *x_i_*, *y_i_*, *z_i_*) is obtained. However, if the obtained minimum distance *D_min_* < 2 pixels, we can use *P_i_* as the estimated value Pout of the cutter holder. If *D_min_* > 2 pixels, we can change Δ*k* to Δ*k*/2, then take it and *P_i_* as the initial values. Finally, all the above processes are repeated until Δ*k* is equal to 0.1 mm. Finally, we can obtain the pose result *P_out_*.

The step-accelerating method can replace the “violent search” mode in a specific range and greatly improve the pose solving rate.

To verify the accuracy and robustness of the proposed OPnP + DM method in a strong noise environment, we compare it with the most advanced PnP methods through analysis of simulation results. Moreover, we also carried out experimental tests on the motion platform built in the laboratory to verify the proposed method’s feasibility.

## 3. Simulation Results

Due to the harsh industrial environment of high humidity and high pressure, the cutter holder image collected contains intense noise. To meet the requirement of positioning accuracy of 1 mm for the cutter holder pose measurement, this paper focuses on the influence of noise intensity on the method under the fixed number of feature points.

**Figure 6:**
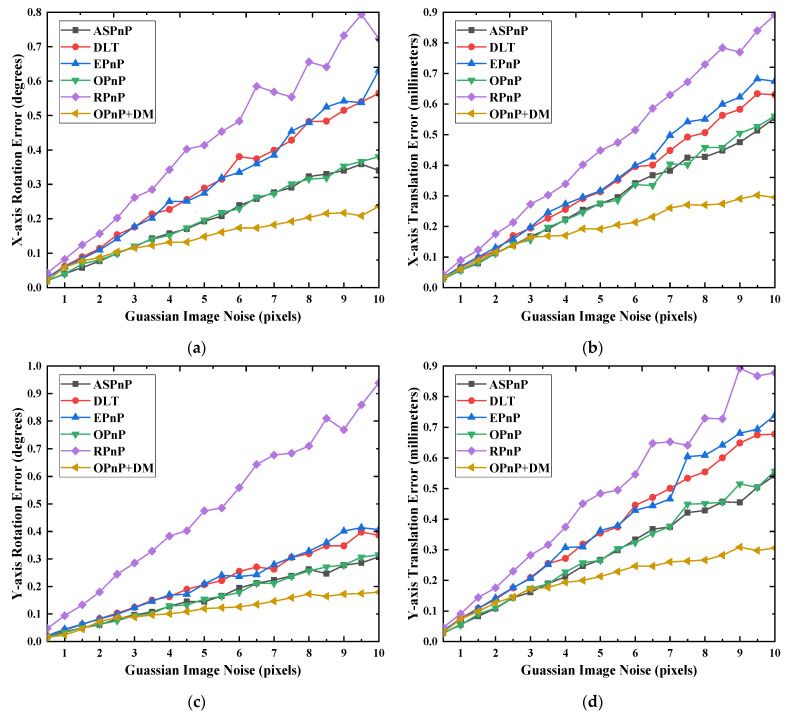

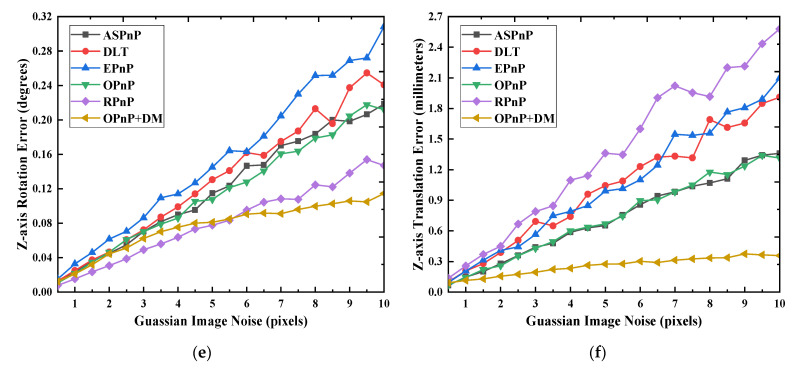
Accuracies of all methods when noise level changes. (**a**) The rotation error in the *X*-axis direction; (**b**) The movement error in the *X*-axis direction; (**c**) The rotation error in the *Y*-axis direction; (**d**) The movement error in the *Y*-axis direction; (**e**) The rotation error in the *Z*-axis direction; (**f**) The movement error in the *Z*-axis direction.

In the simulation experiment, a virtual perspective camera with a resolution of 4112 × 3008 pixels is synthesized, and its focal length and pixel size are 8.5 mm and 3.45 μm, respectively. The movement range of the cutter holder in the *X* and *Y* directions is [−90, 90] mm, the movement range in the depth direction is [740, 760] mm, and the rotation range in the three degrees of freedom directions is [−60, 60] degrees. A cutter holder pose *p* is randomly generated in this space range, and we can obtain the image coordinates corresponding to 20 feature points on the cutter holder surface under this pose state by the camera projection model. After that, different levels of Gaussian noise are added to the image feature points, and 500 test data sets are generated for each noise level. Then the cutter holder pose *p_estimation_* of each test is calculated using PnP methods or the OPnP + DM method. The error between *p_estimation_* and the truth value *p* is calculated finally. We can solve the standard deviation of the 500 error values obtained, which is used to represent the solution accuracy of the methods. The variance of interference noise is successively increased from 0.5 to 10 pixels (the step is 0.5 pixels), and the solving accuracy of each degree of freedom direction of the monocular measurement methods is shown in Figure 6.

The RPnP method has the worst performance among these PnP methods. In most degrees of freedom directions, its accuracy decreases fastest with increased noise intensity. Compared to the other PnP methods, one significant advantage of the RPnP method is that it has higher solution accuracy in the *Z*-axis rotation direction. Among the remaining PnP methods, the OPnP method has the best performance in all the directions. Its accuracy is always the highest one among all PnP methods except in the *Z*-axis rotation direction. However, it can be seen from Figure 6 that the accuracy of the OPnP + DM method is higher than that of PnP methods, especially in the depth direction. In addition, with the increase of noise intensity, the solution accuracy of all PnP methods decreases, and the relationship between them is approximately linear. As shown in Figure 6f, in the *Z*-axis direction, the RPnP method can guarantee 1 mm measurement accuracy only when the variance of interference noise is less than 4 pixels. Among the PnP methods, the OPnP and ASPnP methods can also reach the same accuracy on the premise that the variance of interference noise is less than 7 pixels. However, the OPnP + DM method can guarantee an accuracy of 0.5 mm even if the noise variance is 10 pixels. In a word, its measurement accuracy in the depth direction under strong noise is far better than that of the PnP method. According to the results in Figure 6, the OPnP + DM method has better robustness because its accuracy changes less than the PnP methods when the noise increases. It also has high solving accuracy under strong noise, which makes it more suitable for the disc cutter changing operation site with strong interference. The DM method relies on the initial pose provided by the OPnP method, it is only suitable for a small search range if the initial pose is given randomly. We set the search range of the cutter holder pose in the rotation direction and the movement direction to be [−0.5, 0.5] degrees, [−5, 5] mm, respectively. The average times required by the DM method and OPnP + DM method to search the cutter holder pose is 3.24 s and 19.32 s, respectively. Thus, it can be concluded that the solving speed of the OpnP + DM method is 5.96 times that of the DM method.

## 4. Experiments and Results

We set up a motion platform in the laboratory to verify that the OpnP+DM method has higher measurement accuracy than the PnP methods under complex conditions and can meet the positioning accuracy of 1 mm required by the visual system on the disc cutter changing robot in the moving directions. The input of the experiment is the cutter holder images collected by the vision system, and the output is the actual pose of the cutter holder measured by the pose estimation methods. We calculate the pose of the cutter holder through the pose estimation methods of the visual system and compare it with the pose recorded by the motion platform.

**Figure 7:**
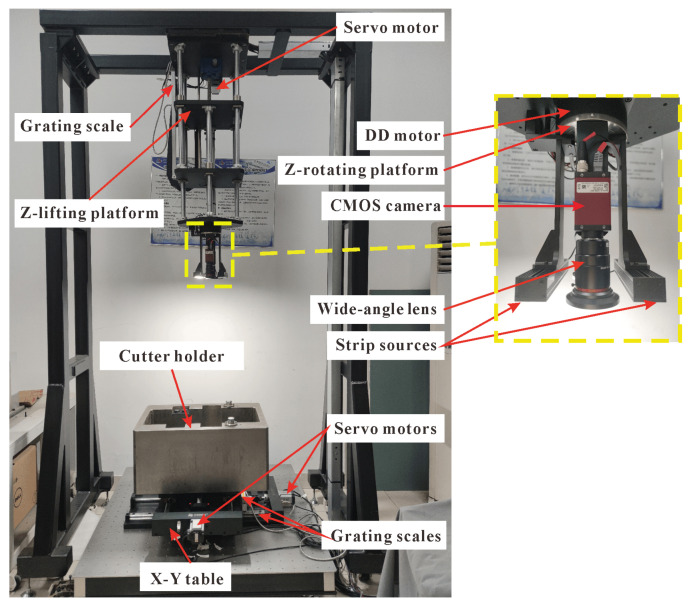
Visual system and four-axis motion platform.

The four-axis motion platform used in the experiments is shown in Figure 7. The platform is equipped with high-precision grating rulers in the *X*-axis, *Y*-axis, and *Z*-axis directions, which enables the platform to achieve a movement accuracy of 0.01 mm in these three directions. Moreover, a DD motor with rotation accuracy of 0.02° is installed in the rotation direction of *Z*-axis of the platform. The monocular vision system of the platform uses the Allied Vision’s Manta G-1236 camera, which has the same resolution, pixel size, and focal length as the virtual camera in the above simulation experiment. Before the measurement, we first use Zhang Zhengyou’s [35] calibration method to calibrate the monocular camera, in which a 7 × 7 circular calibration board with a machining accuracy of 0.01 mm is used. After calibration, we can get the internal parameters of the camera: the focal length obtained is 8.56 mm, pixel size obtained is (3.45, 3.45) μm, and the principal point coordinate obtained is (2190.03, 1427.46) pixels.

We should first establish the pose template library of the cutter holder according to the internal parameters of the camera. Within the motion range of the cutter holder described in the simulation experiment, the step length of displacement direction is set as 0.1 mm, and the step length of rotation direction is set as 0.1° to change the pose of the cutter holder. By substituting the internal parameters of the calibrated camera into the projection model, the 20 image coordinates corresponding to the 20 feature points of the cutter holder in each pose state in the moving space can be obtained. The image coordinates of all feature points under each pose are stored, so the pose template library of the cutter holder is constructed, which means each template in the template library contains only the image coordinates of 20 feature points.

**Figure 8:**
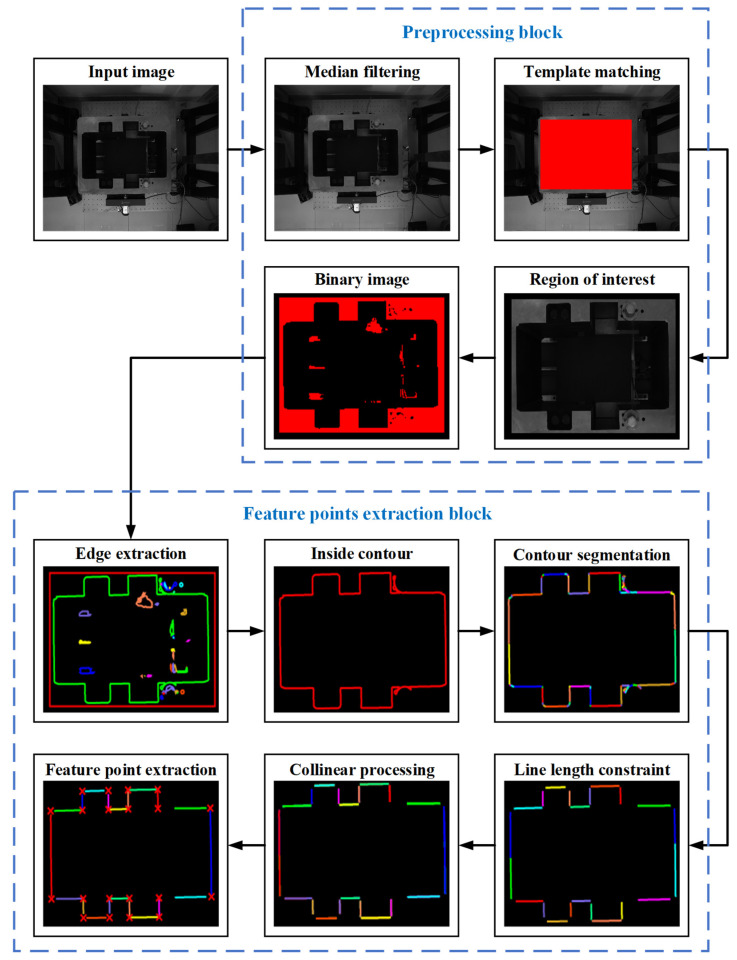
Image processing flow diagram.

After setting up the pose template library, the vision system collects the actual image of the cutter holder and processes it with the image processing method mentioned above, and the results of image processing are shown in Figure 8. The preprocessing block includes denoising, template matching, and binarization. For median filtering, the field shape is square and the convolution kernel size is 3. The feature points extraction block contains edge extraction, inside contour selection, contour segmentation, feature line segments screening, collinear processing, and intersection calculation. In the feature line segments screening, we set a length threshold of 130 pixels according to the image size of the bolt and other interference structures, and only the part with a length greater than it will be screened out. Finally, the obtained 20 feature points are shown in a small graph at the lower left of Figure 8, represented by red crosses.

**Figure 9:**
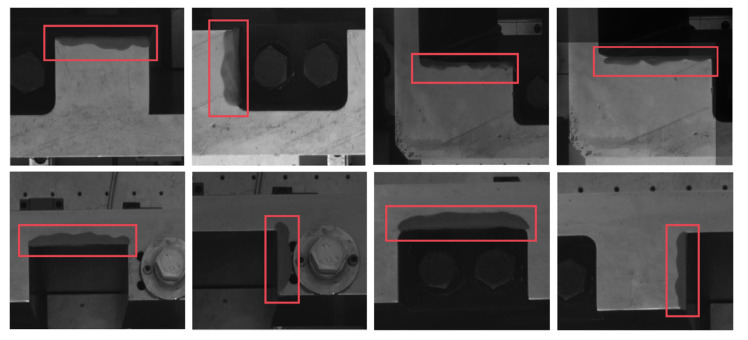
Partial images of the contaminated parts of the cutter holder. The red boxes show the various contamination conditions of the cutter holder.

There will be a piece of washing equipment at the construction site to wash the disc cutter that needs to be replaced and its holder. However, the cutter holder cannot be completely cleaned, and there will always be residual silt to block the feature that needs to be detected. In order to simulate the state that the silt covers the cutter holder, we use soil to block the inside contour of the cutter holder randomly in the measurement experiments. We randomly select one or more straight-line segments on the inside contour of the cutter holder for occlusion in the experiments. Figure 9 shows some screenshots of the occlusion of the straight-line segments, showing the state of occlusion of the inside contour in the experiments. We block the whole straight-line segment when adding soil to ensure that the OPnP+DM method still has good measurement accuracy under such extreme conditions.

The contaminated cutter holder images are processed by the above image processing method. We can obtain the DT image of the binary image containing only 20 feature points, similar to Figure 4. The OPnP method is used to calculate the initial pose of the cutter holder, which along with the initial rotation step value of 3.2° and the initial translation step value of 3.2 mm are the inputs of the DM method. We can obtain the template with the smallest distance from the actual cutter holder in the pose template library by the DM method. The step acceleration method is used in the search process at the same time. The obtained template pose is taken as the final pose of the actual cutter holder. In the experiment, 100 cutter holder images were collected. The error in the directions of various degrees of freedom can be obtained by comparing the calculated cutter holder pose with the actual pose recorded by the platform.

**Figure 10:**
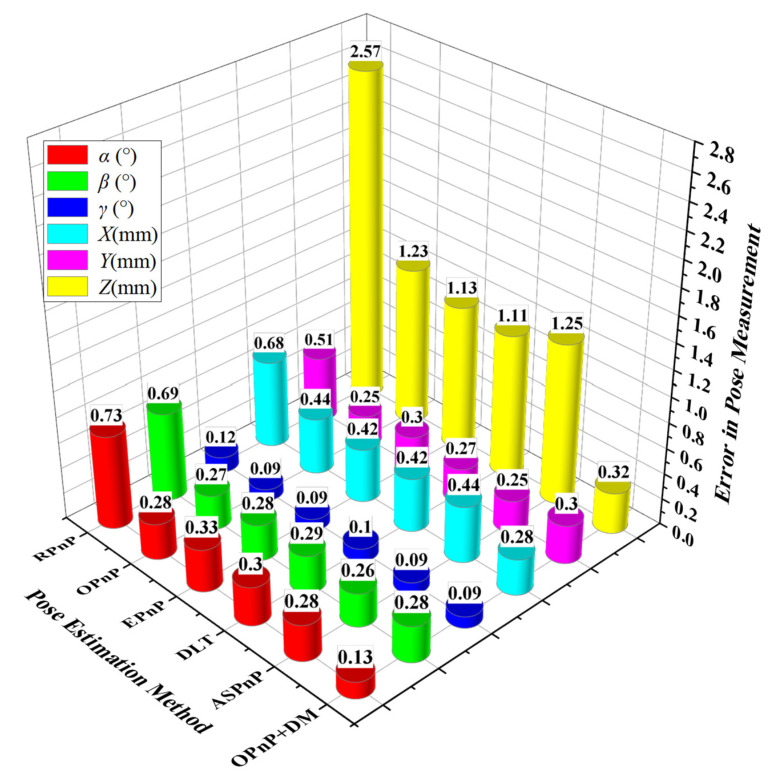
Standard deviation of pose error.

The PnP methods are also used to solve the pose of the cutter holder in the experiments. We calculated the standard deviation of errors in the direction of 6 degrees of freedom for 100 groups of poses respectively, and the results are shown in Figure 10. The rotation and translation errors of the cutter holder pose obtained by the OPnP + DM method in *X*, *Y*, and *Z* axes are 0.13°, 0.28°, 0.09°, 0.28 mm, 0.30 mm, and 0.32 mm, respectively. Similar to the simulation results, the RPnP method has the worst accuracy among several PnP methods, especially in the depth direction, with an error of 2.57 mm. The errors of other PnP methods in the depth direction are also more than 1 mm, which cannot meet the requirements of practical visual measurement accuracy. In the *X*-axis rotation, *X*-axis translation, and *Z*-axis rotation directions, the OPnP + DM method has the highest solving accuracy compared with the PnP methods. Besides this, its accuracy in the *Y*-axis rotation direction and *Y*-axis translation direction are not much different from that of other PnP methods except the RPnP method with poor accuracy. The experimental results show that all the pose estimation methods’ rotation accuracy and translation accuracy are similar in both *X*-axis and *Y*-axis directions, and the rotation accuracy of the *Z*-axis direction is the highest one, which can reach 0.12°. Among the translational degrees of freedom, the translation accuracy in the *Z*-axis direction is the lowest one, reaching 2.57 mm, far exceeding the accuracy of 1 mm required for engineering applications. The experimental results are the same as the simulation results when the variance of Gaussian noise is greater than 8 pixels. In a word, the OPnP + DM method presented in this paper has the highest accuracy, especially in the depth direction. It also has good robustness under strong interference because it can still achieve high precision and meet the actual accuracy requirement of 1 mm when the noise suddenly increases.

## 5. Conclusions

This paper presents a method to solve the pose of the cutter holder on the shield machine based on its surface feature points. The pose template library of the cutter holder is established offline in the method, which improves the detection efficiency. The OPnP method with high accuracy among PnP methods is selected to estimate the initial pose of the cutter holder. Then, the highly robust DM method is used for an accurate pose solution. At the same time, we propose a step-size acceleration strategy to speed up the screening of the minimum distance. Finally, the template pose corresponding to the minimum distance is taken as the final estimated pose of the cutter holder. The simulation results show that the OPnP + DM method proposed in this paper has higher solution accuracy and robustness than several mainstream PnP methods. Especially in the depth direction, the accuracy of the proposed method is much higher than other methods. When the interference noise is strong, only the OPnP + DM method can meet the positioning requirement of measurement accuracy in the depth direction of the visual system. Furthermore, many experiments are also carried out on a built four-axis motion platform; the maximum error of the OPnP + DM method is 0.28° in the direction of rotation and 0.32 mm in the direction of translation, which proves that the OPnP + DM method can obtain the precise pose of the cutter holder under strong interference. The measurement accuracy of the OPnP + DM method is lower than that of PnP methods in some degrees of freedom directions when the interference noise is weak. Moreover, the solving speed of the OPnP + DM is slower than the PnP methods, so we will optimize the method to improve its performance in future work.

## References

[1] Li L., Sun S., Wang J., Song S., Fang Z., Zhang M. (2020). Development of compound EPB shield model test system for studying the water inrushes in karst regions. Tunn.Undergr. Space Technol..

[2] Chen X., Wang Y., Chen L., Ji J. (2020). Multi-Vehicle Cooperative Target Tracking with Time-Varying Localization Uncertainty via Recursive Variational Bayesian Inference. Sensors.

[3] Gadwe A., Ren H. (2019). Real-Time 6DOF Pose Estimation of Endoscopic Instruments Using Printable Markers. IEEE Sensors J..

[4] Yin F., Chou W., Wu Y., Dong M. (2020). Relative pose determination of uncooperative known target based on extracting region of interest. Meas.Control..

[5] Zhang Z., Zhang S., Li Q. (2016). Robust and Accurate Vision-Based Pose Estimation Algorithm Based on Four Coplanar Feature Points. Sensors.

[6] Teng X., Yu Q., Luo J., Wang G., Zhang X. (2019). Aircraft Pose Estimation Based on Geometry Structure Features and Line Correspondences. Sensors.

[7] Liu Z., Liu X., Duan G., Tan J. (2019). Precise pose and radius estimation of circular target based on binocular vision. Meas. Sci. Technol..

[8] Pasqualetto Cassinis L., Fonod R., Gill E. (2019). Review of the robustness and applicability of monocular pose estimation systems for relative navigation with an uncooperative spacecraft. Prog. Aerosp. Sci..

[9] Zhao K., Sun Y., Zhang Y., Li H. (2021). Monocular Visual Position and Attitude Estimation Method of a Drogue Based on Coaxial Constraints. Sensors.

[10] Zhai G., Zhang W., Hu W., Ji Z. (2020). Coal Mine Rescue Robots Based on Binocular Vision: A Review of the State of the Art. IEEE Access.

[11] Liu W., Wu S., Wu Z., Wu X. (2019). Incremental Pose Map Optimization for Monocular Vision SLAM Based on Similarity Transformation. Sensors.

[12] Lixia F., Wang T., Shen Y., Wang P., Wu M. (2021). Parallel collaborative planning for the coupled system of underground heavy-load robot. Adv. Mech. Eng..

[13] Lourakis M., Terzakis G. (2021). A Globally Optimal Method for the PnP Problem with MRP Rotation Parameterization. Proceedings of the 2020 25th International Conference on Pattern Recognition (ICPR).

[14] Přibyl B., Zemčík P., Čadík M. (2017). Absolute pose estimation from line correspondences using direct linear transformation. Comput. Vis. Image Underst..

[15] Barone F., Marrazzo M., Oton C.J. (2020). Camera Calibration with Weighted Direct Linear Transformation and Anisotropic Uncertainties of Image Control Points. Sensors.

[16] Li S., Xu C., Xie M. (2012). A Robust O(n) Solution to the Perspective-n-Point Problem. IEEE Trans. Pattern Anal. Mach. Intell..

[17] Sharma S., Ventura J., D’Amico S. (2018). Robust Model-Based Monocular Pose Initialization for Noncooperative Spacecraft Rendezvous. J. Spacecraft Rockets.

[18] Ghosh S., Ray R., Vadali S.R.K., Shome S.N., Nandy S. (2016). Reliable pose estimation of underwater dock using single camera: A scene invariant approach. Mach. Vis. Appl..

[19] Gong X., Lv Y., Xu X., Wang Y., Li M. (2021). Pose Estimation of Omnidirectional Camera with Improved EPnP Algorithm. Sensors.

[20] Zheng Y., Sugimoto S., Okutomi M. (2013). ASPnP: An Accurate and Scalable Solution to the Perspective-n-Point Problem. IEICE Trans. Inf. Syst..

[21] Zheng Y., Kuang Y., Sugimoto S., Astrom K., OkutomI M. (2013). Revisiting the PnP Problem: A Fast, General and Optimal Solution. Proceedings of the 2013 IEEE International Conference on Computer Vision.

[22] Yan K., Zhao R., Tian H., Liu E., Zhang Z. (2018). A high accuracy method for pose estimation based on rotation parameters. Measurement.

[23] Holzer S., Hinterstoisser S., Ilic S., Navab N. (2009). Distance transform templates for object detection and pose estimation. Proceedings of the 2009 IEEE Conference on Computer Vision and Pattern Recognition (CVPR).

[24] Shih F.Y., Wu Y.-T. (2004). The efficient algorithms for achieving Euclidean distance transformation. IEEE Trans. Image Process..

[25] Zheng T., Duan Z., Wang J., Lu G., Li S., Yu Z. (2021). Research on Distance Transform and Neural Network Lidar Information Sampling Classification-Based Semantic Segmentation of 2D Indoor Room Maps. Sensors.

[26] Bai L., Yang X., Gao H. (2017). Improved chamfer matching method for surface mount component positioning. IET Image Process.

[27] Cardarilli G.C., Di Nunzio L., Fazzolari R., Nannarelli A., Re M., Spano S. (2020). $N$ -Dimensional Approximation of Euclidean Distance. IEEE Trans. Circuits Syst. II.

[28] Sun Y., Li S., Wang Y., Wang X. (2021). Fault diagnosis of rolling bearing based on empirical mode decomposition and improved manhattan distance in symmetrized dot pattern image. Mech. Syst. Signal Process.

[29] Yu Q., Wei H., Yang C. (2017). Local part chamfer matching for shape-based object detection. Pattern Recognit..

[30] Shih F.C., Mitchell O.R. (1992). A mathematical morphology approach to Euclidean distance transformation. IEEE Trans. Image Process..

[31] Liu C., Guo W., Hu W., Chen R., Liu J. (2021). Real-Time Model-Based Monocular Pose Tracking for an Asteroid by Contour Fitting. IEEE Trans. Aerosp. Electron. Syst..

[32] Hu J., Liu S., Liu J., Wang Z., Huang H. (2021). Pipe pose estimation based on machine vision. Measurement.

[33] Wang Z. (2021). An algorithm for ATM recognition of spliced money based on image features. Multimed Tools Appl.

[34] Kukelova Z., Bujnak M., Pajdla T., Forsyth D., Torr P., Zisserman A. (2008). Automatic Generator of Minimal Problem Solvers. Computer Vision – ECCV 2008.

[35] Zhang Z. (1999). Flexible camera calibration by viewing a plane from unknown orientations. Proceedings of the Seventh IEEE International Conference on Computer Vision.

